# Predictive Value of Cardiac Magnetic Resonance Feature Tracking after Acute Myocardial Infarction: A Comparison with Dobutamine Stress Echocardiography

**DOI:** 10.3390/jcm10225261

**Published:** 2021-11-12

**Authors:** Filipa X. Valente, José Gavara, Laura Gutierrez, Cesar Rios-Navarro, Pau Rello, Manel Maymi, Ruben Fernandez-Galera, José V. Monmeneu, Augusto Sao-Aviles, Maria P. Lopez-Lereu, M. Teresa Gonzalez-Alujas, David Moratal, Hug Cuellar, José Barrabés, Imanol Otaegui, Artur Evangelista, Ignacio Ferreira, Vicente Bodi, José Rodriguez-Palomares

**Affiliations:** 1 Cardiology Department, Hospital Universitari Vall d’Hebron, 08035 Barcelona, Spain; lauraguga@gmail.com (L.G.); paurellosabate@gmail.com (P.R.); m.maymi.b@gmail.com (M.M.); rubenfdezgalera@gmail.com (R.F.-G.); saoavilesaugusto@gmail.com (A.S.-A.); teresagonzalu@gmail.com (M.T.G.-A.); jabarrabes@vhebron.net (J.B.); iotaegui@vhebron.net (I.O.); arturevangelistamasip@gmail.com (A.E.); nachoferreira@secardiologia.es (I.F.); jfrodriguezpalomares@gmail.com (J.R.-P.); 2 Centro de Biomateriales y Ingeniería de Tejidos, Universitat Politècnica de Valencia, 46022 Valencia, Spain; Jose_4_6_90@hotmail.com (J.G.); dmoratal@eln.upv.es (D.M.); 3 Instituto de Investigación Sanitaria INCLIVA, 46010 Valencia, Spain; cesar_rios1@hotmail.com (C.R.-N.); Vicente.Bodi@uv.es (V.B.); 4 Unidad de Resonancia Magnética Cardiovascular, Exploraciones Radiológicas Especiales (ERESA), 46015 Valencia, Spain; jmonmeneu@eresa.com (J.V.M.); mplopezl@ascires.com (M.P.L.-L.); 5 Radiology Department, Hospital Universitari Vall d’Hebron, 08035 Barcelona, Spain; hugcuellar@gmail.com; 6 Cardiology Department, Hospital Clínico Universitario de Valencia, 46010 Valencia, Spain; 7 Centrode Investigación Biomédica en Red—Cardiovascular (CIBER-CV), 28029 Madrid, Spain; 8 Medicine Department, Facultad de Medicina y Odontología, Universidad de Valencia, 46010 Valencia, Spain

**Keywords:** cardiac magnetic resonance feature-tracking, myocardial deformation, acute ST-segment elevation myocardial infarction, low-dose dobutamine stress echocardiography, speckle-tracking echocardiography

## Abstract

In acute ST-segment elevation myocardial infarction (STEMI) late gadolinium enhancement (LGE) may underestimate segmental functional recovery. We evaluated the predictive value of cardiac magnetic resonance (CMR) feature-tracking (FT) for functional recovery and whether it incremented the value of LGE compared to low-dose dobutamine stress echocardiography (LDDSE) and speckle-tracking echocardiography (STE). Eighty patients underwent LDDSE and CMR within 5–7 days after STEMI and segmental functional recovery was defined as improvement in wall-motion at 6-months CMR. Optimal conventional and FT parameters were analyzed and then also applied to an external validation cohort of 222 STEMI patients. Circumferential strain (CS) was the strongest CMR-FT predictor and addition to LGE increased the overall accuracy to 74% and was especially relevant in segments with 50–74% LGE (AUC 0.60 vs. 0.75, *p* = 0.001). LDDSE increased the overall accuracy to 71%, and in the 50–74% LGE subgroup improved the AUC from 0.60 to 0.69 (*p* = 0.039). LGE + CS showed similar value as LGE + LDDSE. In the validation cohort, CS was also the strongest CMR-FT predictor of recovery and addition of CS to LGE improved overall accuracy to 73% although this difference was not significant (AUC 0.69, *p* = 0.44). Conclusion: CS is the strongest CMR-FT predictor of segmental functional recovery after STEMI. Its incremental value to LGE is comparable to that of LDDSE whilst avoiding an inotropic stress agent. CS is especially relevant in segments with 50–74% LGE where accuracy is lower and further testing is frequently required to clarify the potential for recovery.

## 1. Introduction

In ischemic heart disease, cardiac magnetic resonance (CMR) allows assessment of cardiac structure and function and provides parameters with important prognostic implications [1] such as left ventricular volumes and ejection fraction (LVEF), infarct size and presence of microvascular obstruction (MVO). At a segmental level, infarct transmurality predicts functional recovery and a late-gadolinium enhancement transmural extension (LGE) of <50% is widely accepted as the cut-off for recovery. Nevertheless, in acute ST-segment elevation myocardial infarction (STEMI) there may be extensive myocardial edema which is quantified with STIR or T2 mapping sequences and used for the estimation of the myocardial area-at-risk [2]. Myocardial edema translates into an increase of the extracellular space and a larger distribution volume of gadolinium-based contrasts, which can result in LGE overestimating the true extension of myocardial necrosis and hence underestimating the potential for functional recovery [3]. Therefore, especially in segments with intermediate LGE (between 50 to 75%), it may be advisable to perform a dobutamine stress test as it shows a higher accuracy for prediction of recovery [4].

CMR feature-tracking (CMR-FT) algorithms allow analysis of myocardial deformation in routinely acquired cine images, in a process analogous to speckle-tracking echocardiography (STE) and have already shown clinical prognostic relevance [5,6,7]. Nevertheless, the additional value of feature-tracking for prediction of recovery and its ability to further risk stratify segments with intermediate LGE, thus avoiding the need for dobutamine stress testing, remains to be clearly established.

It was our purpose to analyze the ability of CMR-FT to predict functional recovery and to analyze whether it offers additional value to LGE, as compared to low-dose dobutamine stress echocardiography (LDDSE) and STE. Subsequently, we sought to validate the study group results in an external validation cohort.

## 2. Materials and Methods

The study group included patients enrolled in a prior double-blind randomized clinical trial in which STEMI patients were randomized to 4.5 mg of adenosine or saline intracoronary injection immediately before percutaneous coronary intervention [8]. After successful primary PCI, all patients presented with grade 3 TIMI flow. The primary endpoint was relative infarct size, and the study protocol is published elsewhere [8].

For the present study, patients were included if they had a CMR and a LDDSE performed on the same day, 3–7 days after STEMI. A second CMR was performed at 6-months and the primary endpoint was recovery of segmental myocardial function, defined as a decrease in wall motion score ≥1. Apart from the LDDSE wall motion response, all remaining conventional and myocardial deformation parameters were analyzed as continuous variables and optimal cut-offs for prediction of recovery were identified.

Subsequently, the cut-offs of the best predictors were applied to an external validation cohort of 222 patients from a prospective STEMI registry of another tertiary hospital; similar inclusion and exclusion criteria were used, basal CMR was performed 7 ± 2 days after STEMI and a follow-up CMR was performed at 6-months, as indicated per local study-protocol. Similar acquisition protocols and software were used, and data analysis was performed by local personnel. In this cohort, LGE extension was classified semi-quantitatively in three groups: 0%, 1–49% and ≥50%. Dobutamine stress echocardiography was not performed in this cohort.

All procedures complied with the Declaration of Helsinki and were approved by the local Ethics Committee. All patients gave their written informed consent.

### 2.1. CMR Examination

CMR was performed in a clinical 1.5 T whole-body MR scanner (Magnetom Symphony, Siemens). The study protocol included 2D-balanced steady-state free precession (b-SSFP) cine images, T2-weighted short-tau inversion-recovery (STIR) images and inversion-recovery gradient echo sequences for LGE analysis. All data were stored in DICOM format and analysis was made off-line according to standard recommendations [9]. Segmental analysis was performed according to the AHA 16-segment model and a wall motion score (WMS) was attributed to each segment (normokinesis 1, hypokinesis 2, akinesis 3, dyskinesis 4). Myocardial deformation analysis was performed with Tissue Tracking (CVI42^®^, version 5.2.1, Circle Cardiovascular Imaging, Calgary, AB, Canada) using standard b-SSFP cine images. One cardiologist dedicated to CMR (F.V.) performed all CMR-FT analyses.

For the 6-month CMR only 2D-b-SSFP cine images were acquired, and no intravenous contrast was administered.

### 2.2. Low Dose Dobutamine Stress Echocardiography (LDDSE)

Echocardiography was performed using a commercially-available standard ultrasound scanner (Vivid 9, GE Vingmed Ultrasound AS, Horten, Norway) with a 3.5 MHz transducer. A standard LDD protocol was performed with image acquisition at rest and at 5 and 10 mcg/kg/min in 3-min stages [10]. As with CMR, a WMS was attributed to each segment according to the AHA 16-segment model. A dysfunctional segment was considered viable if there was at least a 1-grade improvement with dobutamine infusion.

Longitudinal STE strain and strain-rate (SR) analysis were performed at rest and at peak LDDSE (EchoPAC^®^ PC 11.0, GE Vingmed, Horten, Norway). One experienced echocardiographer (L.G.) performed all STE analyses.

Both intra and interobserver variability of CMR-FT and STE have been reported previously for our center [11]. Further details on the technical aspects of CMR and LDDSE acquisition and CMR-FT and STE analysis are provided as Supplemental Material.

### 2.3. Statistical Analysis

Continuous variables are presented as mean ± standard deviation. Qualitative variables are presented as frequencies and percentages. Continuous variables were tested for normal distribution using the Shapiro–Wilk test and, accordingly, comparisons were performed with Student’s *t*-tests or the Mann–Whitney *U*-test. Receiver operator characteristics (ROC) curves were used to define the optimal cut-off values using the Youden index, and to assess the ability of different strain parameters to predict recovery. Comparisons between ROC curves for different strain and SR parameters were made with the method of DeLong et al. [12]. All statistical tests were two-sided and a *p*-value < 0.05 was considered statistically significant. All analyses were performed with SPSS 19.0 (SPSS Inc., Chicago, IL, USA).

## 3. Results

**Figure 1:**
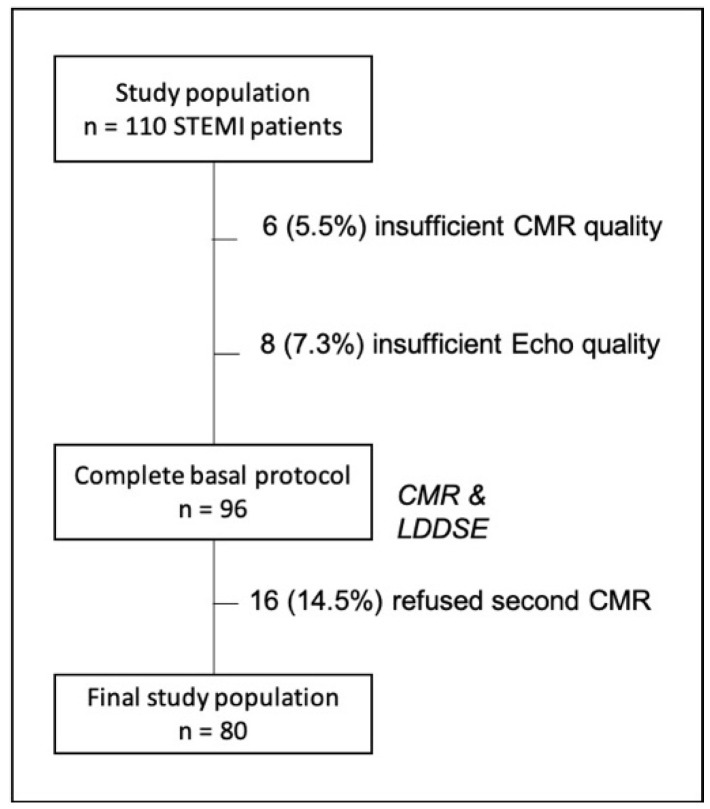
Flow chart showing the study population and reasons for patient exclusion.

One hundred and ten STEMI patients underwent both CMR and LDDSE and were enrolled in the study. Six patients (5.5%) were excluded because of inadequate images for CMR-FT analysis due to severe cardiac and/or respiratory motion artifacts and eight (7.3%) due to sub-optimal echocardiographic window, deemed insufficient for STE analysis. At the six-month follow-up, 16 patients (14.5%) refused to receive a second CMR and were excluded from the final analysis (Figure 1). The baseline characteristics of the final study population (*n* = 80) are shown in Table 1. Mean interval between baseline echocardiography and CMR was 6.7 ± 2 h and no complications related to any of the imaging procedures occurred. Neither fatal nor non-fatal adverse events occurred during the 6-month follow-up period.

At baseline, 897 segments were normokinetic (70.1%), 85 were hypokinetic (6.6%), 293 were akinetic (22.9%) and five were dyskinetic (0.4%). As the number of dyskinetic segments was small and there were no significant differences compared to akinetic segments, they were included in the akinetic category.

Segments with higher wall motion score showed significantly larger LGE and edema extension, as well as lower myocardial salvage index (MSI), CMR-FT, and LDDSE parameters (Table S1). There were no significant differences concerning wall thickness and only akinetic segments had a significantly higher prevalence of MVO and IMH.

### 3.1. Segmental Functional Recovery

At follow-up, 222 out of 383 segments with baseline wall motion abnormalities (58.0%) showed functional recovery. All analyzed CMR and STE parameters showed significant differences between segments that recovered and those that did not recover, and all were predictors of recovery with exception of wall thickness and change from resting to LDD strain and strain rate (Table 2).

**Figure 2:**
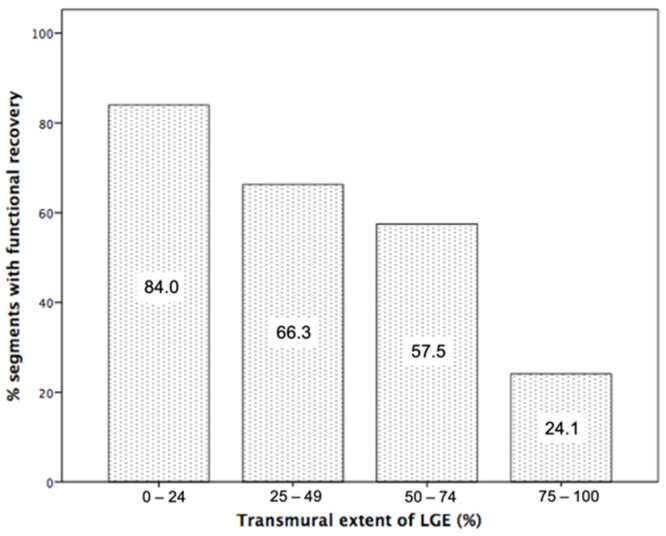
Percentage of segments showing functional recovery according to % of late gadolinium enhancement transmurality (LGE).

LGE transmurality was the strongest of the conventional CMR parameters for prediction of recovery (Table 3). Using a cut-off of 50% for LGE transmurality, it showed 64% of accuracy, 57% of sensitivity, and 73% of specificity. Functional recovery decreased with increasing infarct transmurality and occurred in 84% of segments with <25% infarct transmurality, 66% with 25–49% transmurality, 57% with 50–74% transmurality, and 24% with ≥75% transmurality (Figure 2).

CMR-FT analysis of radial and circumferential strain was correctly performed in all but 5 segments (feasibility 98.7%) and in all but 4 segments (99.0%) for longitudinal strain (LS). Of the three CMR strain parameters, circumferential strain (CS) showed the strongest predictive value with 73% of accuracy, 68% sensitivity and 81% specificity for a cut-off of −10.0% (Table 4).

LDDSE showed an overall accuracy of 67%: 139 segments were accurately predicted to recover yielding a sensitivity of 63%, while absence of recovery was correctly predicted in 117 segments, yielding a specificity of 73%.

STE analysis was correctly performed in 381 segments at rest (99.5%) and 360 segments at LDD (93.9%). LS at rest was the strongest STE predictor with 66% of accuracy, 70% sensitivity and 60% specificity for a cut-off of −8.6% (Table 4). Analysis of the change from rest to LDD strain and strain rate showed no significant differences between segments that recovered and those that did not, and it was not a predictor of functional recovery (ΔLS AUC 0.47, *p* = 0.287 and ΔLSR AUC 0.46, *p* = 0.164).

### 3.2. Multiparametric Prediction of Functional Recovery

**Figure 3:**
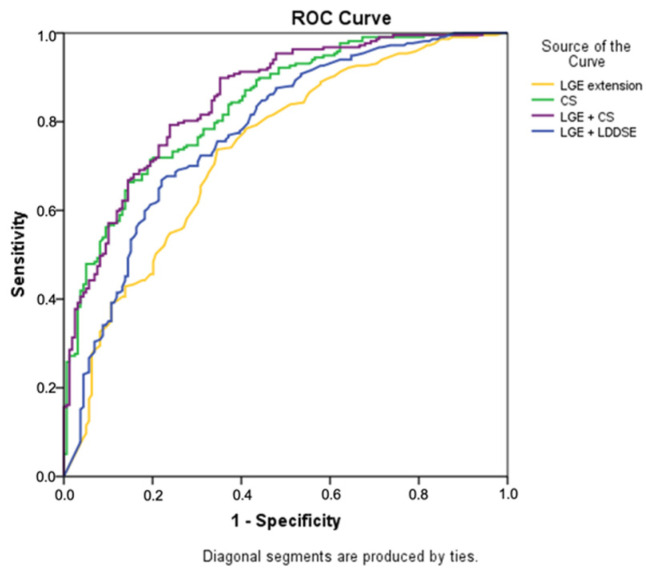
ROC curve analysis for prediction of functional recovery comparing LGE transmurality, CS and the combination of LGE plus CS and LGE plus LDDSE. CS, circumferential strain. LDDSE, low dose dobutamine stress echocardiography. LGE, late gadolinium enhancement transmurality.

**Figure 4:**
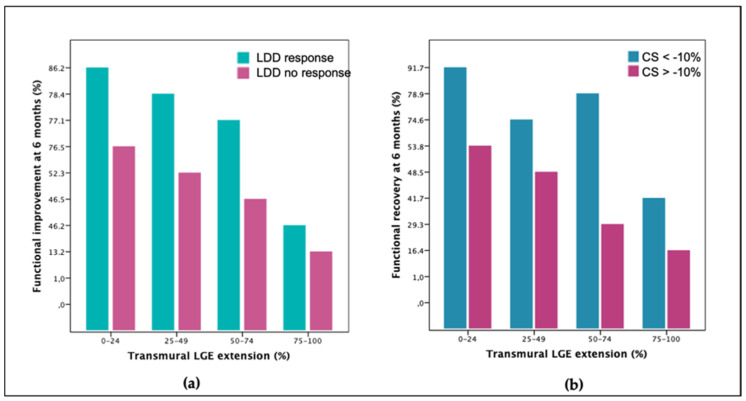
Functional recovery at follow-up comparing each quartile of LGE transmurality and respective subgroups split according to the result of LDDSE (**a**) and of CS (**b**). CS, circumferential strain. LDD, low-dose dobutamine. LGE, late-gadolinium enhancement transmurality.

Adding LDDSE wall motion analysis to LGE transmurality resulted in an increased overall accuracy for prediction of recovery to 71% (Table 5 and Figure 3). Figure 4a shows how LDDSE response complemented LGE information throughout the range of infarct transmurality. LDDSE was particularly useful in the subgroup with 50–74% of LGE where AUC improved from 0.60 to 0.69 (*p* = 0.039). Adding either rest or LDD strain to LGE did not improve prediction of recovery.

Adding CS analysis to LGE transmurality increased overall accuracy for prediction of recovery to 74% (AUC 0.68 vs. 0.73, *p* = 0.017, Table 5 and Figure 3) and complemented LGE information throughout the range of infarct transmurality (Figure 4b). In the subgroup with 50–74% LGE, accuracy improved from 62% to 77% (AUC 0.60 vs. 0.75, *p* = 0.001). The combination of LGE plus CS showed a similar magnitude of improvement as the combination of LGE plus LDD for prediction of functional recovery (*p* = 0.095 overall, *p* = 0.090 for the 50–74% LGE subgroup).

### 3.3. External Validation Cohort

The external validation cohort included 222 STEMI patients with similar clinical characteristics (Table S2). Of the total of 3552 segments, 2603 were normokinetic (73.3%) at baseline CMR, while 949 (26.7%) had wall motion abnormalities: 271 (7.6%) were hypokinetic, 655 (18.4%) were akinetic and 23 (0.6%) were dyskinetic. At follow-up, 367 (38.7%) segments showed functional recovery. As with the study group, segments with functional recovery showed higher CMR-FT strain values and lower LGE transmurality (Table 3).

LGE was predictive of functional recovery with an AUC of 0.68 (95% CI 0.642–0.714) and a cut-off of 50% showed 69% of accuracy, 59% sensitivity, and 77% specificity.

Of the CMR-FT parameters, CS was the best predictor with an AUC of 0.64 (95% CI 0.603–0.677), showing 41% sensitivity and 78% specificity for a cut-off of −10%. Adding CS analysis to LGE improved overall accuracy to 73%, with 60% of sensitivity, 75% of specificity and an AUC of 0.69, although this improvement was not statistically significant (*p* = 0.44).

## 4. Discussion

The current study shows that CMR-FT CS provides additional prognostic value to LGE transmurality for prediction of segmental functional recovery in acute STEMI. The incremental value is comparable to that of LDDSE which suggests that CS could be a useful alternative to LDD in the acute setting.

Cardiac magnetic resonance has an indisputable role in the assessment of myocardial infarction and viability, in particular, through the analysis of LGE. Nevertheless, in acute myocardial infarction LGE can overestimate infarct size and several studies have shown functional improvement in a significant number of segments with ≥50% of infarct extension [13,14]. In our study, LGE also underestimated functional recovery, especially in the subgroup with 50–74% of infarct transmurality. These findings reinforce the need to improve prediction of myocardial viability and functional recovery early after STEMI.

While LGE represents loss of cellular integrity and translates the anatomical extent of the infarcted myocardium, dobutamine stress studies evaluate the contractile reserve of the remaining non-infarcted myocardium and have been recommended for better clarification of the potential for functional recovery in segments with intermediate infarct transmurality [15,16,17]. Dobutamine stress echocardiography is an established technique for the assessment of myocardial viability, with high sensitivity (79–83%) and specificity (82–86%) [18]. As expected, in our study population, addition of LDDSE information to LGE improved overall accuracy to predict recovery. On the other hand, addition of the more quantitative speckle-tracking analysis did not increase its prognostic value, which underlines the value of LDDSE even though it is dependent on the operator’s subjective analysis. Novel echocardiographic measures of left ventricular function such as non-invasive myocardial work have the advantage of integrating afterload into strain measurement and have recently been shown to be a potential marker of segmental myocardial viability and stunning after STEMI [19,20]. These parameters were not analyzed in our study but could have improved the predictive value of LDDSE for functional recovery.

To the best of our knowledge, this is the first study that compares the predictive value of LGE, LDDSE with speckle-tracking and CMR-FT in the same patient population.

All analyzed CMR-FT parameters were significant predictors of functional recovery. CS was the strongest and, most importantly, its incremental value in addition to LGE was similar to that of LDDSE. Previous studies have established the value of global CS to predict outcome in ischemic cardiomyopathy [5,21,22,23]. However, at a segmental level, although CMR-FT was predictive of functional improvement, results have been controversial with respect to its added value to LGE [24,25]. In a study of STEMI patients with a concurrent chronic total occlusion (CTO), CS was also a strong predictor of wall thickening recovery, both in the infarcted and the CTO territory, although it improved independently of whether a CTO-PCI was performed or not [26]. One of the main issues of CMR-FT is the lack of standardization with respect to the optical flow algorithm used, the endocardial-only versus endocardial and epicardial feature-tracking and the imaging planes that are tracked, amongst other variables. In a large meta-analysis of healthy subjects, mean CS was −23.0% (−24.3 to −21.7%) [27], although the heterogeneity of strain analysis also led to difficulties in defining reference values for normal CMR strain [28,29]. We included both endocardial and epicardial feature-tracking as well as whole-heart instead of a single-short axis slice analysis, which may explain the different results as compared to other groups [24,25]. In our study, CS improved the value of LGE along the whole spectrum of infarct transmurality, and in particular in the subgroup with 50–74% of infarct transmurality where it showed the highest added value to discriminate functional improvement. This incremental value is probably related to the mid-myocardial and subepicardial location of circumferential myofibers, as opposed to the longitudinal orientation of subendocardial myofibers [30]. Due to this disposition, CS would be mostly affected in extensive myocardial infarctions while LS, as well as conventional parameters that follow the subendocardial-to-subepicardial ischemic wave-front such as myocardial edema, would have less discriminative power between subendocardial and transmural infarctions.

Finally, the prognostic value, sensitivity and specificity of a variable varies with the prevalence of the disease, through multiple factors and interactions [31]. Although the same inclusion and exclusion criteria were used, the prevalence of functional recovery was significantly lower in the validation cohort (38.7% vs. 58% study cohort, *p* = 0.001). CS was similarly validated as the best CMR-FT predictor of functional improvement in the validation cohort; however, it showed lower sensitivity which impacted negatively in the overall accuracy.

Two main clinical implications can be derived from our study. Firstly, as CS can be easily quantified from routinely-acquired cine images without the need of further pulse sequences and with a user-friendly software interface, strain analysis can be performed in all segments with LGE in order to increase the accuracy for prediction of functional recovery, thus avoiding the need of further imaging studies with inotropic agents. This ideal “one-stop shop” can be especially relevant in the acute patient where the use of pharmacologic stressors may be undesirable. In these patients, accurate prediction of functional recovery is of the utmost importance for optimization of patient management in the short and long-term, both for medical and device treatment, as it will reflect on left ventricular ejection fraction, ventricular remodeling, and on prognosis. The clinical application of CS in this context is in agreement with the increasingly observed paradigm change in the evaluation of cardiomyopathies and valvular heart disease shifting the focus from load and volume-dependent left ventricular wall thickening and ejection fraction to earlier and subclinical markers of cardiac damage, such as myocardial strain [32].

Secondly, in patients with severe renal insufficiency or a contraindication for gadolinium administration, CMR is mostly used for quantification of left ventricular volumes and systolic function; however, CS could also be used as an alternative to LGE quantification for prediction of functional recovery. We believe this to be an area of great potential for CMR-FT and further studies evaluating functional recovery and/or its relationship with native T1/T2 mapping in this subgroup of patients could be of interest.

### Limitations

We believe the lack of standardization of the multiple commercially available CMR-FT software is the most important limitation when our study is compared to that of other groups [33]. The same issue has been debated regarding the widely available speckle-tracking algorithms and has not denied its value but rather resulted in a joint initiative to overcome this problem [34,35]. We initially demonstrated the predictive value of CS in a study population where it was compared to the longstanding value of LDDSE, and then it was further validated in a large population from an independent center. Furthermore, albeit with variable prognostic power, other groups have also shown the predictive value of CS. Therefore, we believe our results add to the overall data that supports the value of segmental CS in this setting.

## 5. Conclusions

Addition of CMR-FT CS improves the value of LGE alone for prediction of functional recovery after STEMI. The incremental value of CS is similar to that of LDDSE and obviates the need of inotropic agents in the acute setting. Improvement of the predictive value was particularly relevant in segments with 50–74% of infarct transmurality, where LGE often generates uncertainty and further testing is frequently required to clarify the potential for recovery.

## Figures and Tables

**Table 1:** Baseline characteristics of the study population (*n* = 80).

**Clinical Characteristics**	
Age, years	59.2 ± 12.5
Male sex, *n* (%)	70 (87.5)
Hypertension, *n* (%)	33 (41.3)
*Diabetes mellitus*, *n* (%)	10 (12.5)
Dyslipidemia, *n* (%)	21 (26.3)
Smoking, *n* (%)	57 (71.3)
Sinus rhythm, *n* (%)	80 (100)
**Angiographic Findings**	
Culprit artery, *n* (%)	
RCA	34 (42.5)
LAD	35 (43.8)
LCx	11 (13.8)
Multivessel disease, %	28 (35.0)
Time to reperfusion, min	207.9 ± 65.4
**CMR Parameters**	
LVEDV, mL/m^2^	83.3 ± 18.5
LVESV, mL/m^2^	42.5 ± 23.6
LVEF, %	50.7 ± 9.6
LV mass, g/m^2^	71.2 ± 15.3
Relative infarct mass, %	21.0 ± 14.1
Relative edema mass, %	33.1 ± 12.9
Myocardial salvage index, %	42.6 ± 17.2
Microvascular obstruction, *n* (%)	39 (48.8)

LAD, left anterior descending artery. LCx, left circumflex artery. LVEDV, left ventricular end-diastolic volume. LVEF, left ventricular ejection fraction. LVESV, left ventricular end-systolic volume. RCA, right coronary artery.

**Table 2:** CMR and LDDSE parameters according to functional recovery at 6-months.

	Parameter	Functional Recovery	No Functional Recovery	*p*-Value
	*n* (%)	222 (58.0)	161 (42.0)	-
Conventional CMR parameters	Wall thickness, mm	8.6 ± 1.7	8.3 ± 1.7	0.087
	LGE, %	41.5 ± 24.9	62.8 ± 25.3	0.001
	Edema, %	64.3 ± 29.4	83.5 ± 23.0	0.001
	MSI, %	38.8 ± 28.7	28.7 ± 24.3	0.001
	MVO, *n* (%)	34 (15.3)	58 (36.0)	0.001
	IMH, *n* (%)	10 (4.5)	19 (12.3)	0.006
CMR-FT parameters	RS, %	22.9 ± 17.1	8.3 ± 12.0	0.001
	CS, %	−13.5 ± 6.8	−3.6 ± 8.1	0.001
	LS, %	−11.2 ± 5.1	−6.3 ± 5.2	0.001
LDDSE	LS_rest_, %	−11.4 ± 4.9	−7.3 ± 5.4	0.001
	LS_LDD_, %	−13.3 ± 6.0	−8.8 ± 6.4	0.001
	LSR_rest_, s^−1^	−0.80 ± 0.3	−0.66 ± 0.4	0.001
	LSR_LDD_, s^−1^	−0.98 ± 0.5	−0.76 ± 0.4	0.001
	ΔLS, %	−2.31 ± 8.1	−1.40 ± 4.4	0.219
	ΔLSR, s^−1^	0.22 ± 2.4	0.09 ± 1.4	0.160

CMR, cardiac magnetic resonance. CS, circumferential strain. IMH, intramyocardial hemorrhage. LDDSE, low dose dobutamine stress echocardiogram. LGE, late-gadolinium enhancement transmurality. LS, longitudinal strain. LSR, longitudinal strain rate. MSI, myocardial salvage index. MVO, microvascular obstruction. RS, radial strain. Δ, change from basal to 6-month CMR.

**Table 3:** Conventional CMR and prediction of functional recovery at follow-up.

	AUC	95% CI	Cut-Off	Sens	Spec	OR	95% CI	*p*-Value
Wall thickness (mm)	0.557	0.498–0.616	8.0	60%	47%	1.114	0.984–1.261	0.088
LGE (%)	0.647	0.582–0.703	50%	57%	73%	0.967	0.958–0.976	0.001
Edema (%)	0.640	0.584–0.696	80%	61%	74%	0.972	0.963–0.981	0.001
MSI (%)	0.606	0.545–0.665	29%	59%	61%	1.014	1.006–1.023	0.001
MVO (*if present*)	0.604	0.545–0.662	-	-	-	0.321	0.197–0.523	0.001
IMH (*if present*)	0.539	0.479–0.599	-	-	-	0.339	0.153–0.752	0.008

CMR, cardiac magnetic resonance. IMH, intramyocardial hemorrhage. LGE, late-gadolinium enhancement transmurality. MSI, myocardial salvage index. MVO, microvascular obstruction.

**Table 4:** CMR-FT and LDDSE strain for prediction of functional recovery at follow-up.

	AUC	95% CI	Cut-Off	Sens	Spec	OR	95% CI	*p*-Value
RS	0.701	0.647–0.755	16%	77%	63%	1.081 per 1%	1.059–1.102	0.001
CS	0.725	0.673–0.778	−10.0%	68%	81%	1.229 per −1%	1.173–1.284	0.001
LS	0.675	0.620–0.730	−10.0%	72%	63%	1.203 per −1%	1.147–1.262	0.001
LS_rest_	0.710	0.657–0.762	−8.6%	70%	60%	1.165 per −1%	1.114–1.219	0.001
LS_LDD_	0.690	0.635–0.746	−10.0%	68%	56%	1.129 per −1%	1.085–1.174	0.001
LSR_rest_	0.636	0.578–0.694	−0.62 s^−1^	70%	55%	3.26 per −1 s^−1^	1.683–6.329	0.001
LSR_LDD_	0.648	0.591–0.706	−0.73 s^−1^	68%	51%	3.79 per −1 s^−1^	2.119–6.803	0.001

CS, circumferential strain. CMR-FT, cardiac magnetic resonance feature tracking. LDD, low-dose dobutamine. LDDSE, low-dose dobutamine stress echocardiography. LS, longitudinal strain. LSR, longitudinal strain rate. RS, radial strain.

**Table 5:** Multiparametric analysis and prediction of functional recovery at follow-up.

	AUC	Accuracy	Sensitivity	Specificity	PPV	NPV
LGE	0.65	64%	57%	73%	74%	55%
LDDSE	0.68	67%	63%	73%	76%	59%
CS	0.72	73%	68%	81%	84%	65%
LGE + LDDSE	0.70	71%	79%	57%	72%	67%
LGE + CS	0.73	74%	84%	61%	75%	73%

CS, circumferential strain. LDDSE, low dose dobutamine stress echocardiography. LGE, late gadolinium enhancement transmurality.

## Data Availability

The data that support the findings of this study are available from the corresponding author upon reasonable request.

## Supplementary Materials

The following are available online at https://www.mdpi.com/article/10.3390/jcm10225261/s1, Document S1: CMR and LDDSE acquisition and analysis, Table S1: CMR and LDDSE parameters according to wall motion score at baseline CMR, Table S2: Baseline clinical characteristics, angiographic findings and conventional CMR parameters of the validation cohort (*n* = 222), Table S3: CMR parameters according to functional recovery at 6 months.
