# Supplementary material for: Predictive Value of Cardiac Magnetic Resonance Feature Tracking after Acute Myocardial Infarction: A Comparison with Dobutamine Stress Echocardiography
Source: J Clin Med. 2021 Nov 12;10(22):5261. doi: 10.3390/jcm10225261 (PMC8624532; doi:10.3390/jcm10225261)
Supplement: Supplementary file 1 [file jcm-10-05261-s001.zip › jcm-1436080-supplementary.pdf]

CMR and LDDSE acquisition and analysis

CMR examination

CMR was performed in a clinical 1.5 T whole-body MR scanner (Magnetom Symphony, Siemens, Erlangen, Germany) using a dedicated cardiac phased-array receiver coil. The standardized study protocol included 2D balanced steady-state free precession (b-SSFP) cine images in 2-, 3- and 4-chamber long-axis planes and a stack of short-axis images from the mitral valve to the apex (TR 3.2 ms, TE 1.5 ms, spatial resolution 1.4x1.4x8.0 mm, retrospective ECG gating, temporal resolution 28–37 msec, 25 phases per cardiac cycle and 7–12 seconds of breath-hold time per image). T2-weighted short-tau inversion-recovery (STIR) sequences were acquired in the same imaging planes (spatial resolution 1.4x1.4x8.0 mm, echo train length 20–40, end-diastolic phase). Finally, 2D segmented inversion-recovery gradient echo sequences images were acquired for LGE analysis 20 minutes after intravenous administration of 0.2 mmol/kg of gadobutrol (spatial resolution 1.4x1.4x8.0 mm, end-diastolic phase). Edema was quantified using the 2-SD technique8 with inclusion of hypointense areas that correspond to intramyocardial hemorrhage (IMH); LGE was quantified using the 5-SD technique8 and inclusion of hypoenhancement zones corresponding to microvascular obstruction (MVO).

CMR-FT analysis

Myocardial deformation analysis was performed with Tissue Tracking (CVI42®, version 5.2.1, Circle Cardiovascular Imaging, Toronto, Canada) using standard b-SSFP cine images. The endocardial and epicardial borders were manually traced in the end-diastolic phase of three long-axis and the short-axis stack. The most basal slice of the short-axis stack to be included was the first that did not present any distortion from the LV outflow tract throughout the cardiac cycle. The anterior insertion of the right ventricle in the short axis-slices was used to define the segments according to the AHA 16-segment model. The software automatically tracks tissue features and generates myocardial deformation curves for longitudinal, circumferential and radial strain. The systolic interval was identified by aortic valve opening and closure as observed in 3-chamber cine images. If wall motion tracking was considered inadequate, minor adjustments were made. If these failed, the segment was excluded from the final analysis.

Low dose dobutamine stress echocardiography (LDDSE)

A standard low-dose dobutamine protocol was performed with image acquisition at rest and at 5 and 10 mcg/kg/min in 3-minute stages. At each stage, images of 3 consecutive cardiac cycles were acquired in 3 standard apical views (4-, 2- and 3-chamber) during breath hold, with a frame rate of 60–80 frames/s and using harmonic imaging. Heart rate and rhythm were continuously monitored, as were blood pressure and the 12-lead ECG at each stage. Side-by-side digital displays were used for wall motion analysis.

Speckle-tracking echocardiography analysis

Longitudinal STE strain and strain-rate analysis were performed at rest and at peak LDDSE (EchoPAC PC 11.0, General Electric Medical Systems, Milwaukee, Wisconsin). A pulse-wave Doppler recording through the LV outflow tract was acquired from a 5-chamber view to identify the systolic interval, defined as the time between aortic valve opening and closure. A region of interest was defined in each view by tracing the endocardium, and the software then automatically tracked each segment. Manual adjustments were made if deemed necessary; however, if tracking quality remained inadequate, the segment was excluded from the analysis. The automatic algorithm generates strain curves for each of the 16 segments.

Table S1. CMR and LDDSE parameters according to wall motion score at baseline CMR.

		Normokinetic	Hypokinetic	Akinetic
	n (%)	897 (70.1)	85 (6.6)	298 (23.3)
Conventional CMR parameters	Wall thickness (mm)	8.2 ±1.7	8.5 ±1.3	8.4 ±1.8
	LGE (%)	5.1 ±12.5	26.8 * ±22.8	57.2 * ± 24.4
	Edema (%)	10.6 ±21.1	49.9 * ±30. 9	78.0 * ± 24.8
	MSI (%)	63.8 ±35.6	54.2 * ±34.5	29.9 * ± 23.0
	MVO, *n* (%)	16 (1.8)	3 (3.5)	89 * + (29.9)
	IMH, *n* (%)	0 (0)	2 (2.3)	29 * + (10.0)
CMR-FT parameters	RS, %	50.5 ±29.8	25.9 * ±21.4	16.4 * ± 37.2
	CS, %	−22.0 ±8.5	−13.2 * ±8.7	−8.2 * ± 8.7
	LS, %	−15.8 ±5.8	−12.8 * ±4.4	−8.0 * ± 5.5
LDDSE	LSrest, %	−16.6 ±5.4	−13.2 * ±5.1	−8.7 * ± 5.3
	LSLDD, %	−18.3 ±6.5	−15.0 * ±6.2	−10.4 * ± 6.3
	LSRrest, s−1	−1.03 ±0.4	−0.9 * ±0.3	−0.7 * ± 0.4
	LSRLDD, s−1	−1.3 ±0.6	−1.1 * ±0.5	−0.8 * ± 0.4

CS, circumferential strain. IMH, intramyocardial hemorrhage. LDDSE, low dose dobutamine stress echocardiogram. LGE, late-gadolinium enhancement transmurality. LS, longitudinal strain. LSR, longitudinal strain rate. MSI, myocardial salvage index. MVO, microvascular obstruction. RS, radial strain. *p-value <0.05 for comparison with normokinetic segments; +p-value <0.05 for comparison with hypokinetic segments

Table S2. Baseline clinical characteristics, angiographic findings and conventional CMR parameters of the validation cohort (*n* = 222).

Clinical characteristics	
Age, years	58.9 ± 11.3
Male sex, *n* (%)	179 (80.6)
Hypertension, *n* (%)	94 (42.3)
Diabetes mellitus, *n* (%)	39 (17.6)
Dyslipidemia, *n* (%)	92 (41.1)
Smoking, *n* (%)	128 (57.7)
Angiographic findings	
Culprit artery, *n* (%)	
RCA	83 (37.4)
LAD	121 (54.5)
LCx	18 (8.1)
Multivessel disease, %	56 (25.2)
Time to reperfusion, min	265.2 ± 219.9
CMR parameters	
LVEDV, mL/m2	80.1 ± 24.7
LVESV, mL/m2	39.6 ± 21.9
LVEF, %	52.4 ± 13.3
LV mass, g/m2	73.1 ± 17.9
Relative infarct mass, %	21.8 ± 15.0
Relative edema mass, %	29.4 ± 17.2
Myocardial salvage index, %	26.9 ± 26.0
Microvascular obstruction, *n* (%)	86 (38.7)

LAD, left anterior descending artery. LCx, left circumflex artery. LV, left ventricle. LVEDV, left ventricular end-diastolic volume. LVEF, left ventricular ejection fraction. LVESV, left ventricular end-systolic volume. RCA, right coronary artery. WMSI, wall motion score index.

Table S3. CMR parameters according to functional recovery at 6 months

Parameter	Functional recovery	No functional recovery	*p* value
n (%)	367 (38.7)	582 (61.3)	-
LGE ≥ 50%	41.1%	76.8%	0.001
RS, %	16.4 ± 20.1	8.2 ± 14.9	0.001
CS, %	−8.2 ± 9.9	−3.6 ± 8.5	0.001
LS, %	−6.9 ± 5.1	−4.9 ± 4.6	0.001

CS, circumferential strain. LGE, late-gadolinium enhancement transmurality. LS, longitudinal strain. RS, radial strain.
